# The decline in reading for pleasure over 20 years of the American Time Use Survey

**DOI:** 10.1016/j.isci.2025.113288

**Published:** 2025-08-20

**Authors:** Jessica K. Bone, Feifei Bu, Jill K. Sonke, Daisy Fancourt

**Affiliations:** 1 Research Department of Behavioural Science and Health, Institute of Epidemiology & Health Care, University College London, London, UK; 2 Center for Arts in Medicine, University of Florida, Gainesville, FL, USA

**Keywords:** Public health, Sociology

## Abstract

Reading has a wide range of benefits for literacy, employment, and health as well as promoting cultural understanding. However, previous monitoring of reading in the US has been inconsistent, with some studies demonstrating large declines over time, and others suggesting engagement has not changed. We measure reading for pleasure and reading with children from 2003 to 2023, using a nationally representative sample from the American Time Use Survey (*n* = 236,270). We found marked declines in the proportion of individuals reading for pleasure daily in the US, with decreases of 3% per year (prevalence ratio = 0.97, 95% confidence interval = 0.97, 0.98, *p* < 0.001). There were disparities across population groups, with widening gaps for those of Black (vs. White) race, with lower education levels and less annual income. Our findings demonstrate the need for more targeted strategies to increase opportunities for reading for pleasure. Monitoring daily reading, and factors influencing reading, will be vital to understand the impacts of future policies.

## Highlights

• Rates of daily reading for pleasure have declined over the past 20 years in the US
• Rates of reading for pleasure differed between population groups
• Disparities across racial groups, levels of education, and income increased over time
• Reading with children daily is less common but has not changed over time

## Graphical abstract

**Figure:**
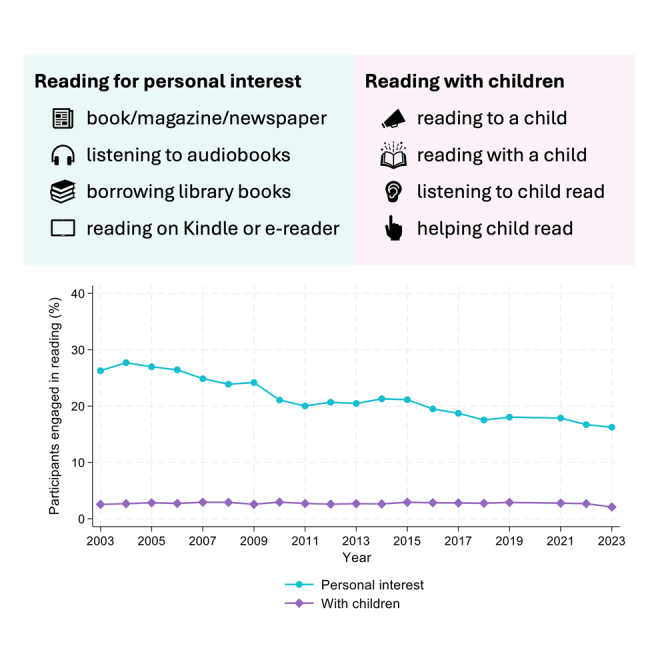

## Introduction

Reading for pleasure, also referred to as leisure reading, comprises any kind of reading done for enjoyment or purposes other than work or school. It is a multidimensional construct with several components, including behavioral, affective, and cognitive elements, which can be accessed through multimodal forms of reading, from print text to e-books and audiobooks.^1^ Reading for pleasure may include reading fiction, non-fiction, magazines, and newspapers, alongside other genres.

Extensive research has explored the benefits of reading, from direct gains in comprehension skills,^2^^,^^3^ vocabulary, logical reasoning, imagination, emotional intelligence, and empathy,^4^ to links with academic achievement, financially rewarding employment, career growth, and involvement in civic life.^5^ Reading may also promote health, reducing stress, anxiety, and depressive symptoms,^6^^,^^7^ supporting better sleep^8^ and slowing cognitive decline in older adults,^9^ as well as increasing longevity.^10^ Impacts of reading can also extend beyond the individual level, with shared experiences in reading being important for bridging cultural gaps, increasing understanding, and building a sense of belonging and identity.^4^

Given the cultural value ascribed to reading, there is a long history of studying its prevalence in the United States (US). Concerns that fewer people may be reading have arisen following declines in reading ability across the population.^11^^,^^12^ Yet, evidence is mixed on whether fewer people are now reading in the US. This has been the subject of several National Endowment for the Arts (NEA) reports, with messages ranging from “reading at risk” (2004)^13^ to “reading on the rise” (2009).^14^ These NEA reports have used data from the nationally representative Survey of Public Participation in the Arts (SPPA), repeated at five-year intervals. Looking across these reports, there appear to have been declines in book reading; in 1992, 61% of adults reported reading a book for pleasure over the last 12 months, but this dropped to 49% by 2022.^13^^,^^15^ Greater declines have been observed among young adults aged 17–18 in the nationally representative Monitoring the Future survey, with the proportion who read a book or magazine every day decreasing from 60% in the late 1970s to 16% by 2016.^16^ Yet, in a set of telephone surveys conducted by Pew Research Center, with a national sample weighted to be representative of US adults (but not randomly recruited), 75% of adults reported reading a book in any format in the last 12 months, and this figure remained similar from 2011 to 2021.^17^

Inconsistent findings are likely due to the limitations of existing data. Research from outside the US is relatively uninformative to understanding US patterns, as there are large cross-country differences in reading, even between high-income Western countries.^18^ Yet, within the US, research has most frequently studied early adolescents, with relatively few studies on adults.^1^ In adult surveys, measures of reading are likely subject to social desirability bias, as many people believe that they should read.^3^ Furthermore, most previous research has asked people about reading habits retrospectively over a one-year period, which is subject to substantial recall biases. Apparent fluctuations in rates of reading may also be a result of combining findings from multiple short-term studies with inconsistent methods.^5^ Additionally, there is a lack of evidence considering the long-term impacts of the COVID-19 pandemic, which may have influenced reading behaviors, as indicated by a dramatic increase in print book sales during and following the pandemic.^19^ Many adults also spend time reading with children in their leisure time, including reading to children, listening to children read, or helping children read. While this is known to be important for children developmentally,^20^^,^^21^^,^^22^ whether levels of reading with children have changed over time remains unclear.

Previous research has also identified a steep social gradient in reading for pleasure in the US, with those who identify as women, White, and as having the highest levels of education and income most likely to read.^4^^,^^5^^,^^13^^,^^14^^,^^17^^,^^18^^,^^23^ But it is less clear whether and how these disparities have changed over time. Some evidence is promising in showing a greater increase in reading in adults with lower income over recent years, resulting in a narrowing gap;^17^ whereas other studies have reported relatively uniform time trends in reading across gender, racial, and socioeconomic groups, with disparities remaining stable over time.^13^^,^^14^^,^^16^ Identifying groups that are least likely to read is vital for informing targeted programmes to increase access to and engagement with reading. Monitoring these disparities over time will not only facilitate evaluation of current policies and interventions^24^ but also support future policy initiatives.

In this study, we used time use survey data, which identifies, quantifies, and classifies people’s behaviors within a specific 24-h window, providing more detail than previous studies, substantially reducing recall bias, and enabling analysis of patterns in behaviors over multiple years. Specifically, we analyzed data from the American Time Use Survey (ATUS), which includes over 10,000 individuals each year from 2003 to 2023, providing nationally representative estimates for an average day over a 20-year period in the US.^25^ We aimed to fully phenotype both reading for pleasure and reading with children as behaviors among adults in the US. Our objectives were to: (1) estimate rates of reading on an average day; (2) determine how long people spent reading, both overall and for different types of reading; (3) explore whom reading was done with; (4) identify where reading was done; (5) examine potential disparities by exploring variation across different subgroups of the population; (6) describe current patterns using the latest available data, and (7) identify the national trends in these measures from 2003 to 2023.

## Results

In total, 236,270 individuals completed the ATUS once between 2003 and 2023 (excluding 2020). We excluded 2020 because of methodological issues due to the COVID-19 pandemic (data collection was paused for part of the year, so weights for 2020 cannot be combined with other years). Participants were aged 15 and over (mean = 45.14, standard deviation [SD] = 18.63). After weighting, 52% were female, 53% were married, 63% were employed, 81% identified as White race, 12% Black, 4% Asian, and 2% identified as American Indian, Alaskan Native, Hawaiian/Pacific Islander, or multiple racial groups (Table 1).

**Table 1:** Characteristics of the sample

Characteristic	Proportion
**Sex**	

Male	48%
Female	52%

**Age**	

15-24 years	17%
25-65 years	67%
66 years and over	16%

**Race**	

White	81%
Black	12%
Asian	4%
Other	2%

**Marital status**	

Married	53%
Widowed/divorced/separated	17%
Never married	30%

Child under 18 in household	39%

**Education**	

High school or less	45%
College	25%
Undergraduate	19%
Postgraduate	11%

**Employment status**	

Employed	63%
Unemployed	5%
Not in labor force	16%
Retired	16%

**Annual family income**	

Less than $30,000	25%
$30,000 - $59,999	28%
$60,000 - $99,999	24%
$100,000 and over	23%

**Metropolitan status**	

Non-metropolitan area	16%
Metropolitan area	84%

Disability that prevents work	4%

	**Mean (SD)**
Household size	2.98 (1.56)
Number of children in household	0.75 (1.13)

*Note.n* = 236,270. Results are weighted and based on 20 imputed datasets.

**Figure 1:**
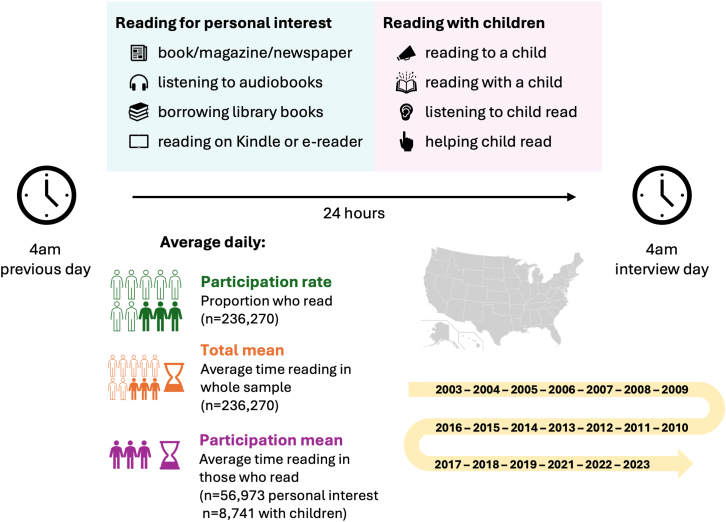
Dataset characterization and measures of reading

ATUS asked participants to recall all their activities over 24 h, beginning at 4 a.m. on the day prior to the interview and ending at 4 a.m. on the day of the interview (Figure 1). Activities were coded using a standard lexicon, verified by two coders. We focused on two reading outcomes: (1) daily reading for pleasure, classified by ATUS as reading for personal interest (e.g., reading a magazine/book/newspaper, listening to audiobooks, reading on a Kindle or other e-reader; Table S1); and (2) daily reading with children (e.g., reading to or with household or non-household children, listening to child read, helping child read; Table S1).

### Reading for pleasure

First, we focused on reading during the average day in 2023, describing current reading practices in the US. Participants spent an average of 16 min (SD = 55) reading for pleasure. Yet only 16% of participants read for pleasure during the diary day. The 1,747 participants who did read for pleasure in 2023 spent an average of 1 h and 37 min (SD = 103, median = 60) reading. Engagement rates in 2023 were lower than the averages across the whole study period (2003–2023), although time spent reading for pleasure was higher in 2023 than for the whole period (Table S4).

**Figure 2:**
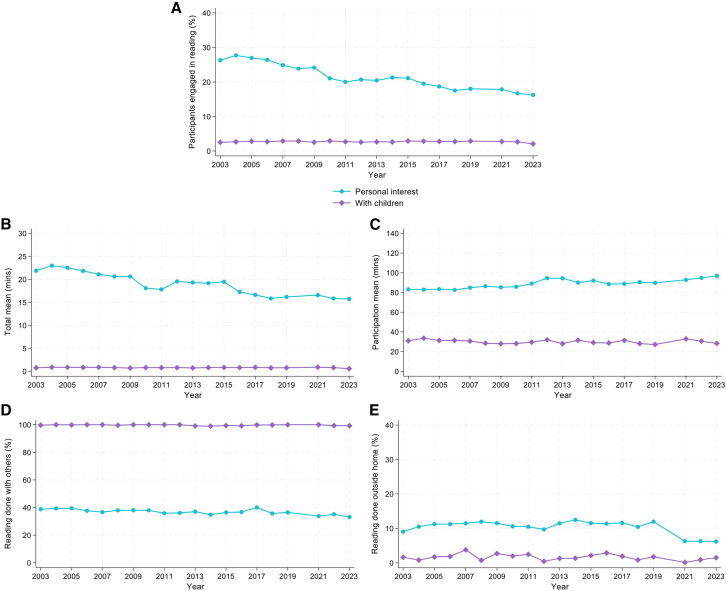
Descriptive statistics showing engagement in reading for personal interest and reading with children from 2003 to 2023, excluding 2020 due to the COVID-19 pandemic *n* = 236,270 in (A) and (B) and *n* = 56,973 for reading for pleasure and *N* = 8,741 for reading with children in (C)-(E). (A) Participation rate: proportion of sample who read. (B) Total mean: average time spent reading in the full sample. (C) Participation mean: average time spent reading just for participants who read. (D) Proportion of participants who read with others. (E) Proportion of participants who read outside the home.

We then examined changes over time. Figures 2A–2C show how these measures fluctuated between 2003 and 2023. Reading for pleasure exhibited a gradual decline following a peak of 28% in 2004, with the lowest rate in 2023. Poisson regression with robust standard errors indicated that this engagement rate decreased by 3% per year from 2003 to 2023 (prevalence ratio [PR] = 0.97, 95% confidence interval [CI] = 0.97, 0.98, *p* < 0.001; Table S5).

For time spent reading for pleasure, there was a decrease in the average time spent reading overall (total mean), which was highest in 2004 (23 min). Using linear regression, the total mean decreased by 0.36 min per year (coef = −0.36, 95% CI = −0.41, −0.32, *p* < 0.001; Table S6). In contrast, the participation time for those who did read for pleasure (participation mean) increased from 1 h to 23 min in 2003 and peaked in 2023. Linear regression models showed that this participation mean increased by 0.62 min per year (coef = 0.62, 95% CI = 0.46, 0.77, *p* < 0.001).

### Reading with children

In 2023, participants spent an average of 1 min (SD = 23) reading with children per day, but only 2% read with children on the diary day. The 200 participants who read with children in 2023 spent an average of 28 min (SD = 19, median = 30) doing so. There was no evidence for changes in reading with children between 2003 and 2023 (Figures 2A–2C; Tables S5 and S6).

### Social context

We also measured the social context and location of reading, as ATUS asked participants whom they were with and where they were for every activity. This additional information was used to provide an indication of the types of reading behaviors engaged in. Given the low frequency of reading in the presence of different people, we created a binary indicator of reading alone vs. with others.

In 2023, most participants who read for pleasure did so alone (67%). In contrast, reading with children was almost always reported as being done in the presence of others (99%; the remaining 1% may have been interacting with children online). When looking at all 20 years of data, as shown in Figure 2D, the proportion of those who read for pleasure in the presence of others decreased very slightly during the study period. In line with this, Poisson regression indicated that the proportion of participants who read with others around decreased by 1% per year (PR = 0.99, 95% CI = 0.99, 1.00, *p* < 0.001; Table S5). Reading with children was nearly always done in the presence of others over the whole period.

### Location

As participants mainly read at home, we measured the proportion of participants who read only in their home/others’ homes vs. outside home. Among those who read for pleasure in 2023, 94% read in their own or others’ homes. Reading with children was done at home even more frequently, with 99% reading in their own or others’ homes. As shown in Figure 2E, the proportion of those who read for pleasure outside the home decreased over the study period, from 9% in 2003 to 6% in 2023. In line with this, Poisson regression indicated that the proportion of participants who read outside the home decreased by 1% per year (PR = 0.99, 95% CI = 0.98, 0.99, *p* < 0.001; Table S5). In contrast, reading with children was nearly always done at home over the whole period.

### The role of individual characteristics

#### Participation rate

**Figure 3:**
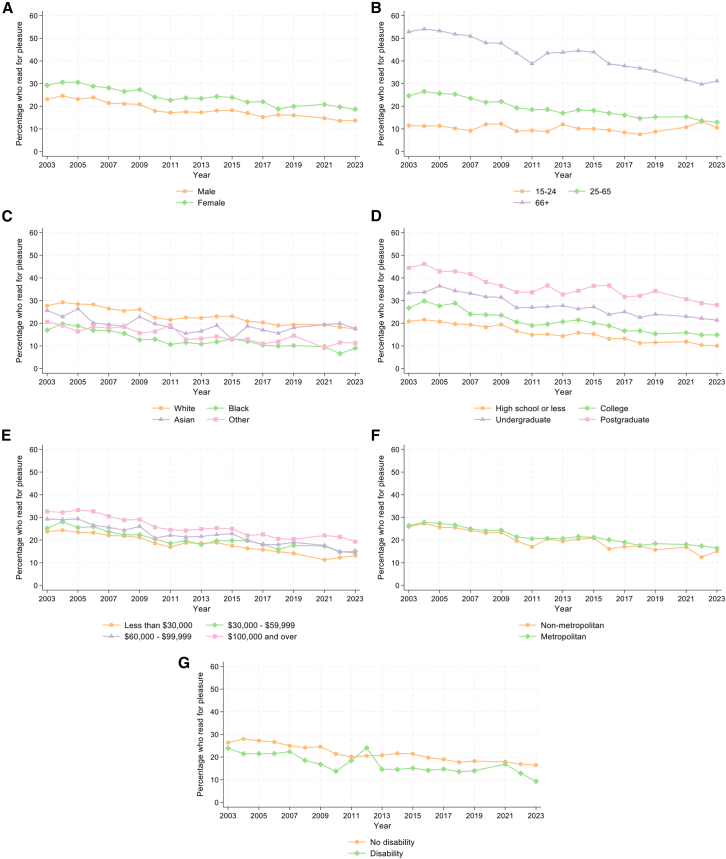
Descriptive statistics showing differential trends in participation rates for reading for pleasure from 2003 to 2023, excluding 2020, stratified by individual characteristics *n* = 236,270. (A) Sex. (B) Age group. (C) Race. (D) Education. (E) Annual family income quartiles. (F) Metropolitan status. (G) Disability status.

**Figure 4:**
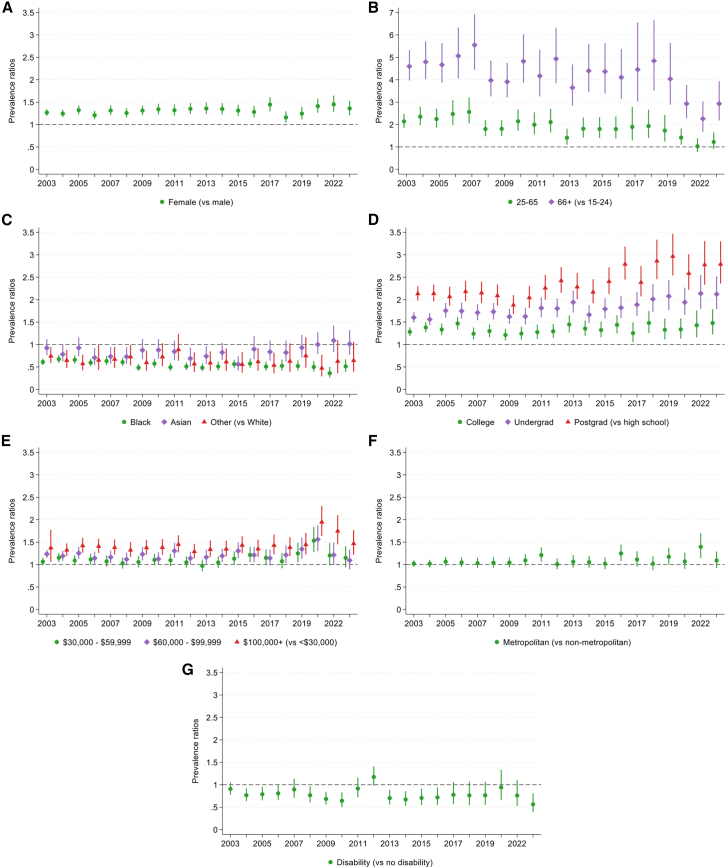
Robust Poisson regressions testing associations between individual characteristics and rates of reading for pleasure, estimated separately for each survey year from 2003 to 2023, excluding 2020. *n* = 236,270 Error bars show 95% CIs and interaction terms from overall regression models are shown in Tabe S7A. (A) Sex. (B) Age group. (C) Race. (D) Education. (E) Annual family income quartiles. (F) Metropolitan status. (G) Disability status.

We then explored the role of individual characteristics in reading for pleasure. Looking at participation rates in 2023 (Figures 3 and 4), robust Poisson regression models showed that females (vs. males), older participants, those with higher education, and the highest level of family income were more likely to read. In contrast, those of Black race (vs. White) and those with a disability (vs. no disability) were less likely to read. However, there was no evidence for differences in overall engagement according to metropolitan status.

The associations between all individual characteristics (except disability status) and engagement rate differed according to survey year (Table S7a). Stratified analyses indicated that the prevalence ratio for gender varied from 2003 to 2023 but did not show a large increase or decrease (Figure 4). In contrast, the prevalence ratios for older participants compared to those aged 15–24 decreased over time, showing a narrowing of the gap between age groups. For race, the prevalence of reading was lower in Black than White participants, and the prevalence ratio got further from 1 (the null value) over time, showing an increasing disparity. By 2023, Black participants had a 49% lower prevalence of daily reading than White participants (PR = 0.51, 95% CI = 0.39, 0.67, *p* < 0.001).

There were stark differences between educational groups, as the prevalence ratios for each level of education (compared to those with a maximum of high school education) all increased over time, showing widening gaps between groups. By 2023, those with postgraduate education had a 2.79 times higher prevalence of daily reading than those with high school or less education (PR = 2.79, 95% CI = 2.36, 3.30, *p* < 0.001). A similar, although less pronounced, pattern can be seen for those with the highest level of income. By 2023, those with the highest income had a 1.47 times higher prevalence of daily reading than those with the lowest income (PR = 1.47, 95% CI = 1.22, 1.76, *p* < 0.001). Although there were no differences in reading by metropolitan area in 2023, prevalence ratios increased throughout the study period, indicating an emerging gap with higher prevalence of reading for those in metropolitan (vs. non-metropolitan) areas.

Given the small group sizes for reading with children, the role of individual characteristics and interactions with time are reported in the supplementary materials (Table S7b).

#### Participation mean

**Figure 5:**
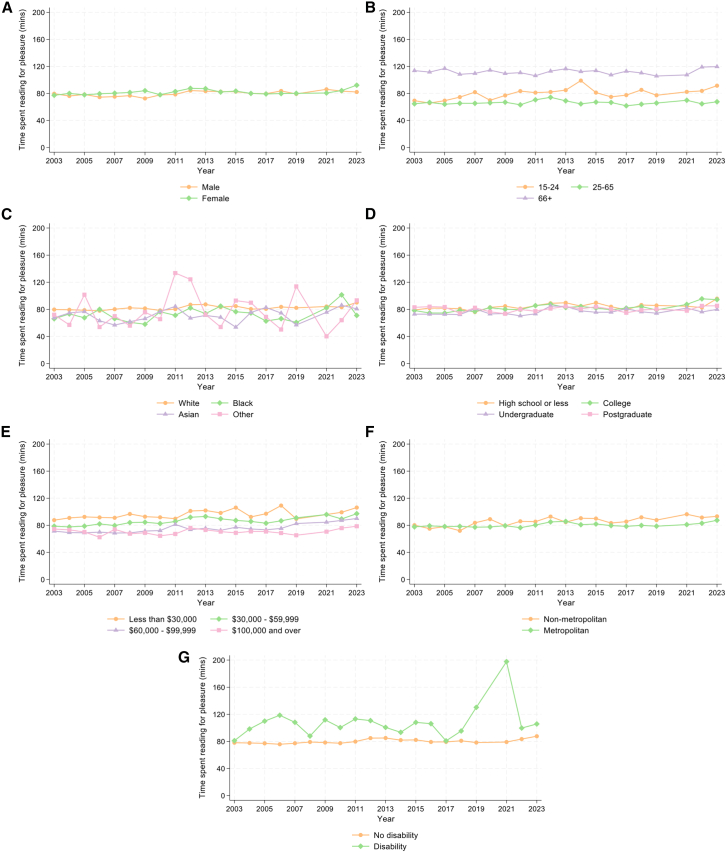
Descriptive statistics showing differential trends in time spent reading for pleasure by those who read (participation mean) from 2003 to 2023, excluding 2020, stratified by individual characteristics *n* = 56,973. (A) Sex. (B) Age group. (C) Race. (D) Education. (E) Annual family income quartiles. (F) Metropolitan status. (G) Disability status.

**Figure 6:**
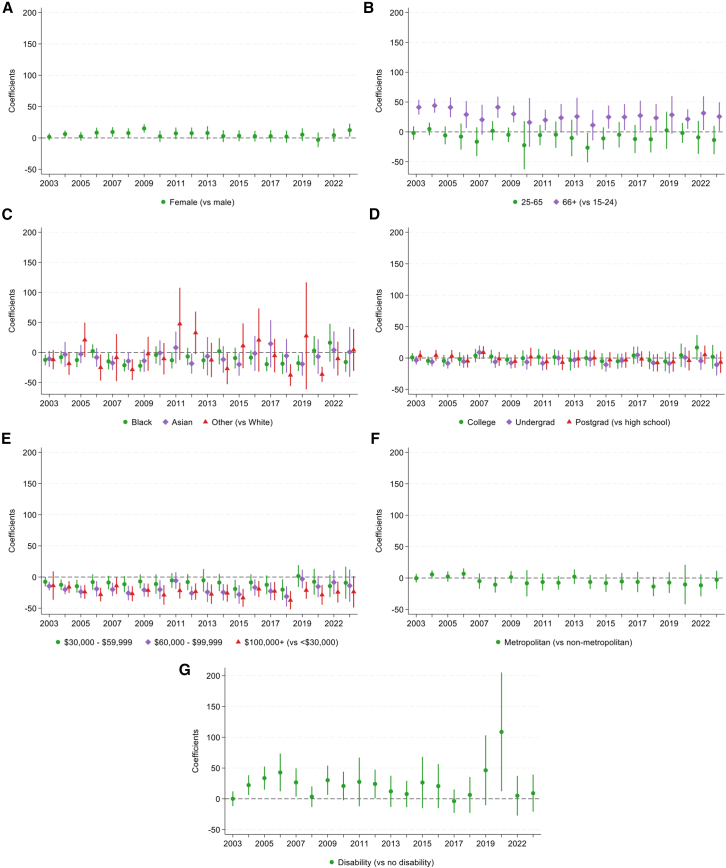
Linear regressions testing associations between individual characteristics and time spent reading for pleasure by those who read - participation mean - estimated separately for each survey year from 2003 to 2023, excluding 2020 *n* = 56,973. Error bars show 95% CIs and interaction terms from overall regression models are shown in Table S7A. (A) Sex. (B) Age group. (C) Race. (D) Education. (E) Annual family income quartiles. (F) Metropolitan status. (G) Disability status.

Time spent reading for pleasure by those who read was less clearly differentiated across population groups (Figures 5 and 6). In 2023, linear regression models showed that females (vs. males) and the oldest participants (vs. those aged 15–24) spent more time reading. Participants of Black (vs. White) race and those with the highest (vs. lowest) income level spent less time reading. There were no differences according to education, metropolitan area, or disability status. Testing whether disparities in time spent reading changed over time, there was only evidence of differences for metropolitan areas (Table S7A). However, there was very little evidence for differences in the time spent reading by those in metropolitan vs. non-metropolitan areas over the study period (Figure 6). Although Figure 5 shows a large increase in time spent reading for pleasure among those with a disability from 2018 to 2021, there was no evidence for linear trends in this relationship across the whole study period.

## Discussion

In a large nationally representative sample of the US population, the proportion who read for pleasure on an average day declined over the last 20 years, from highs of 28% in 2004 to lows of 16% in 2023; a relative decrease of 3% per year. In contrast, there were no changes in reading with children over time. Despite the overall decrease, the amount of time spent reading by those who read for pleasure increased slightly from 1 h to 23 min a day in 2003 to 1 h and 37 min in 2023. Most people reported reading alone and this remained stable over time. A large majority reported reading at home, as opposed to at a library, in the workplace, or other community locations. The proportion who read outside the home decreased over time. Rates of reading varied across population groups, as those who identified as female, of White race, were older, who had higher education, greater annual family income, lived in metropolitan areas, and did not have a disability were more likely to read in 2023. Disparities between population groups increased during the study period for those of Black race, with lower education levels, less annual income, and living in non-metropolitan areas.

The decline in reading for personal interest over the past 20 years in the US is consistent with the most robust previous evidence.^13^^,^^15^^,^^16^ This decline is concerning given earlier evidence for downward trends in reading for pleasure from the 1940s through to the start of our study in 2003, suggesting at least 80 years of continued decline in reading for pleasure.^5^^,^^18^^,^^26^ The extent of the decrease in our study is surprising given that the ATUS definition of reading for personal interest included not only just reading a book, magazine, or newspaper but also listening to audiobooks/books on tapes and reading on a Kindle or other e-reader. It is possible that some digital reading types were missed prior to the inclusion of the Kindle/e-reader example in 2011, or the inclusion of audiobooks alongside tapes in 2021. Yet this is unlikely to explain our findings, particularly as most people who read on an electronic device or listen to audiobooks also read print books.^17^^,^^27^

It has been suggested that declines in daily reading for personal interest are a result of changes in the function of reading and/or the replacement of reading by other media. People are reading less fiction in the US.^15^ They may instead read to fulfill practical needs, using alternative sources of information (online news, websites, and forums).^28^ People have a finite amount of leisure time, and limited cognitive capacity, resulting in an attention economy whereby activities are in competition.^29^ Displacement theory suggests that increasing time and attention spent on other forms of media (e.g., digital media and social media) may replace reading for personal interest.^30^ While this has been debated, declines in reading are correlated with increased use of other digital media in the US.^13^ In Spain, TV watching is negatively associated with book reading, indicating that it may be replacing time spent on reading.^23^ Among young people in particular, digital media use may be replacing reading for personal interest.^16^^,^^31^ Even if other activities are not deliberately chosen to displace reading, distraction by digital devices may still occur,^5^^,^^32^ preventing people from reading. Testing this was beyond the scope of our study but should be explored further in future research across countries.

Although reading with children did not change over time, rates of engagement were surprisingly low, with only 2% of participants reading with children on the average day. Overall, 21% of our sample had a child under 9 years (the age by which most can read independently)^11^ with them during the diary day. So a large majority of those with young children did not read with them. This is concerning given that regular reading during childhood is a strong determinant of reading ability and engagement later in life.^20^^,^^22^ The low rates of reading with children may thus contribute to future declines in reading among adults.

It is unclear whether all types of reading provide equal benefits. While reading with children has wide-ranging positive impacts for the child^20^^,^^22^ and may facilitate bonding with the adult reader,^33^ it might not have the same benefits for the adult as reading for their own personal interest. Within reading for personal interest, there could also be implications of the type of reading material engaged with. For example, reading anything may provide a distraction from stressors in one’s own life and could widen worldviews (among other benefits),^4^ but reading non-fiction may involve fewer opportunities for developing linguistic skills, creativity, imagination, theory of mind, and/or emotion regulation than reading fiction,^34^^,^^35^^,^^36^ and reading the news may even increase stress.^37^ ATUS does not distinguish between the type, genre, or mode of reading, but future research should further explore how these have changed over time, and whether there are differences in the implications for literacy, employment, health, and wellbeing. Given the increasing frequency with which people use other sources of information (online news, websites, and social media) as opposed to print books,^28^ it will also be important to explore whether these should be included in definitions of reading.

Although we could not include data from 2020, we were able to consider the long-term impacts of the COVID-19 pandemic. We expected to find increases in reading from 2019 to 2021 due to the rise in print book sales during the pandemic.^19^ Yet, we did not see an increase in the proportion of participants engaged in reading. There may have been a slowing of the decline in reading, and a slight increase in the amount of time spent reading by those who read, but these differences were very small. Restrictions on movement thus were not accompanied by large increases in reading. Although surprising, this is in line with previous evidence.^15^ An exception was the proportion of people with a disability who read, and the amount of time they spent reading, which dramatically increased in 2021. Although this could be a result of the small group size and/or be influenced by outliers, it suggests that those with a disability were more likely to turn to reading during the pandemic, potentially while isolating at home due to health concerns. However, engagement levels then declined in this group to 2023, suggesting that, even if present, this was not a lasting effect.

There were further disparities in reading over the whole study period, with differences across all the population subgroups tested. The stable gender difference, with females consistently more likely to read than males, was similar to previous evidence.^4^^,^^18^^,^^23^^,^^27^^,^^38^^,^^39^ Although age differences changed over time, this was driven by a narrowing of the gap between age groups, with greater declines in those aged 66 and over (who were still more likely to read than those aged 65 and under). It is possible that this is a cohort effect, with younger generations reading less than previous cohorts.^16^^,^^38^ The most concerning disparities were those that increased over time, with widening gaps between Black and White racial groups, levels of education and income, and metropolitan areas. Participants of Black race, with the lowest education and income levels, and living in non-metropolitan areas were least likely to read in 2003, and showed the steepest decline in reading to 2023, with increasing differences between population groups. These widening gaps contrast with most previous research, which has found stable or decreasing disparities.^13^^,^^14^^,^^16^^,^^17^^,^^27^ Yet, they are in line with inequalities in literacy among young people in the US.^40^^,^^41^ This demonstrates the urgent need for interventions to increase opportunities for reading among these groups.

Our findings have numerous implications for policy and practice. Reading policy often focusses on children and young people,^42^ as reading is fundamental to education, but policymakers must also consider adults, as reading has broader benefits for health and wellbeing, particularly for stress, depression,^6^^,^^7^ and sleep disorders,^8^ which are all on the rise in the US.^43^^,^^44^^,^^45^ The American Library Association is tasked with ensuring equitable access to reading and advocating for adult readers through the Office for Literacy and Outreach Services. An executive order issued by the Biden administration aimed to promote and expand library services to strengthen public health and wellbeing,^46^ but federal priorities changed from 2025. Additionally, relying on libraries may be challenging, given that only 0.3% of our sample reported reading at a library on an average day in 2023, with 0.5% visiting a library for any activity.

Among other initiatives, reading also features heavily in NEA strategic plans. The NEA Big Read supports reading programs around the US, designed to unite communities through books.^47^ From 2006 to 2022, the NEA provided more than $25 million to over 1,800 NEA Big Read programs nationwide, reaching every Congressional district in the US. Over 6 million people have attended an NEA Big Read event.^47^ Celebrity and online book clubs can also impact reading habits. Oprah’s Book Club is perhaps the most prominent example, as it has influenced bestseller lists for years. And yet our findings indicate that this is not enough; the proportion of the population reading for personal interest is still declining. It is possible that these initiatives are only reaching groups who already read, meaning further action is required targeting high-risk groups, particularly where disparities appear to be increasing. Monitoring will be vital to understand the impact of future interventions. Fortunately, the new National Arts Statistics and Evidence-based Reporting Center’s (NASERC) Arts Indicators Project will build on our study, providing regular statistics on reading for pleasure and reading expenditure in the US from 2024 onwards.^48^ Our findings show that initiatives aimed at those of Black race, with lower education levels, less annual income, living in non-metropolitan areas, and with a disability will be important. To support these initiatives, future research must identify the causes of declines in reading and show why disparities are widening.

### Limitations of the study

This study has several strengths. We included over 236,000 people, with weights making estimates nationally representative of an average day in the US. Although response rates declined over time, ATUS measured all daily activities, so there may be less selection bias than surveys explicitly focused on reading. Time use surveys are also less susceptible to recall bias than other surveys that require reporting over longer periods. While overarching categories of reading for personal interest and reading with children remained consistent over time, specific examples of activities within these domains were updated, which is important to keep up with emerging ways of leisure reading.

However, there are some limitations of the ATUS activity classifications. ATUS grouped various types of reading as they are infrequent when measured daily, preventing more detailed investigation, including separating different forms (e.g., print, digital, audio) and genres (e.g., fiction, literature, social purpose). Although they represent contrasting ways of reading, including all of these behaviors allowed us to gain an overview of overall rates of reading in the US, particularly given recent rises in digital and audio engagement, and demographic differences in different forms of reading.^17^^,^^49^ As younger people are more likely to engage in digital/audio reading, while older adults are more likely to read print books,^49^ limiting the definition of reading for pleasure to one type may introduce sociodemographic inequalities. Given the lack of detail on different types and forms of reading in ATUS, we also explored the social context and location of reading to provide further indication of the types of behaviors engaged in. Yet, this does not provide information on why people read, or whether they participated in a book club, which is likely to have greater impacts on reading behaviors,^50^ as well as social and wellbeing outcomes. Building on this, assessing different types and purposes of leisure reading should be a priority for future research.^51^ ATUS did also include some broader reading-related behaviors in the reading for personal interest category, such as borrowing/checking out/returning books and browsing at the library. Although uncommon (0.5% of participants visited a library on the diary day in 2023), so unlikely to influence our findings, future research should separate these behaviors from reading as they are likely to have different effects on cognition, health, and wellbeing.

In the ATUS data, it is possible that some digital reading was classified under other digital activities, particularly before the inclusion of kindles/e-books (2011) and audiobooks (2021) into the reading examples. Similarly, in earlier years (2003–2006), reading the Bible and scripture were also included in reading for personal interest, but these were re-categorized from 2007 onwards, and grouped with other participation in religious practices (so could not be included in our index). This may mean that we underestimated rates of total engagement, although, as outlined previously, we expect any such misclassifications to have minimal effects on our findings. Further, reading on tablets, computers, or smartphones was not explicitly included in examples, making it unclear whether this behavior would have been classified as reading for personal interest or technology use. Our reading measures are thus unlikely to have included activities, such as reading blogs or the news online, which may be a form of reading for pleasure. Future surveys should aim to capture these types of reading, differentiating them from mindless scrolling.

Additionally, in this study, we focused on the average day. This cannot be compared with monthly or annual prevalence rates, as it typically leads to much lower estimates of engagement. Differences between daily and longer-term estimates of reading may partially be driven by the uneven distribution of reading throughout the year, with some mainly reading during holidays.^18^ However, ATUS collects data for 97–98% of days annually, including throughout the summer when people are likely to take vacations. Future studies should explore whether average daily patterns of engagement differ throughout the year. Additionally, the measures of sex (male, female; due to availability in ATUS) and race (White, Black, Asian, Other; due to small numbers in non-White groups) were overly simplistic. This approach conflates experiences across diverse sex, racial, and ethnic groups, which might be problematic as these groups may not have equal opportunities to read. Future research should collect more nuanced data on sex and further explore the role of race and ethnicity in arts participation.

### Conclusions

Overall, we found declines in daily reading over the last 20 years in the US. Although reading with children has not changed, people have become less likely to read for personal interest on the average day. There were disparities in rates of reading according to all individual characteristics assessed (except metropolitan status), with evidence of widening gaps for those of Black race, with lower education levels, less annual income, and living in non-metropolitan areas. This is concerning given the wide-ranging benefits of reading. Our findings thus demonstrate the urgent need for more targeted strategies to increase opportunities for reading for the whole population, and particularly among high-risk groups. Continuing to monitor daily leisure reading levels, as well as the demographic and socioeconomic characteristics influencing reading, will be vital to understand the impacts of future policies.

## Resource availability

### Lead contact

Requests for further information and resources should be directed to and will be fulfilled by the lead contact, Dr Jessica Bone (jessica.bone@ucl.ac.uk).

### Materials availability

This study did not generate new materials.

### Data and code availability

• Data: This study used publicly available ATUS data, available from: https://www.bls.gov/tus/data.htm.
• Code: All original code has been deposited at OSF and is publicly available at https://doi.org/10.17605/OSF.IO/8NBXD as of the date of publication.
• Additional information: All analyses were performed using Stata MP 18.5.^52^ Any additional information required to reanalyze the data reported in this paper is available from the lead contact upon request.

## Acknowledgments

The EpiArts Lab, a National Endowment for the Arts Research Lab at the University of Florida, is supported in part by an award from the National Endowment for the Arts (1936473-38-24). The opinions expressed are those of the authors and do not represent the views of the National Endowment for the Arts Office of Research & Analysis or the National Endowment for the Arts. The National Endowment for the Arts does not guarantee the accuracy or completeness of the information included in this material and is not responsible for any consequences of its use. The EpiArts Lab is also supported by Americans for the Arts, Bloomberg Philanthropies (F024567), the Dharma Endowment Foundation, the Pabst Steinmetz Foundation, and the State of Florida Division of Arts and Culture (24.c.ne.900.834).

## Author contributions

J.K.B., D.F., and F.B. conceptualized the study and developed the analytical plan. J.K.B. performed analyses, with input from D.F. and F.B., and drafted the manuscript. All co-authors contributed to the interpretation and reporting of findings and read and approved the final version of the manuscript.

## Declaration of interests

J.K.S. is a guest editor for the special issue “Transdisciplinary approaches to arts and health: Integrating creative practice in clinical and public health contexts” but was not involved in any parts of the editorial handling of this article.

## STAR★Methods

### Key resources table

**Table:** 

REAGENT or RESOURCE	SOURCE	IDENTIFIER
**Deposited data**		

ATUS 2003–2023 data	US Bureau of Labor Statistics	https://www.bls.gov/tus/data.htm

**Software and algorithms**		

Stata MP 18.5	StataCorp	https://www.stata.com/statamp/
Analytical code	This paper	https://doi.org/10.17605/OSF.IO/8NBXD

### Experimental model and study participant details

The American Time Use Survey (ATUS) is a continuous cross-sectional survey, representing all residents of private households in the US aged 15 and over.^25^ Individuals are randomly selected from a subset of households that have completed their eighth month of interviews for the Current Population Survey (CPS). One individual per household is invited to ATUS two months after completing this CPS interview.

Data collection began in 2003, with data currently available to 2023. We excluded 2020 because of methodological issues due to the COVID-19 pandemic (data collection was paused for part of the year, so weights for 2020 cannot be combined with other years). Approximately 26,400 people were eligible per year, but response rates declined over time (57.8%–35.8%).^25^ Each participant was interviewed only once. ATUS excluded participants who reported fewer than five activities during the diary day and those who reported activities covering fewer than 21 h of the diary day.^25^ This left a total of 236,357 participants, who completed the ATUS once in 2003–2019 or 2021–2023 (Table S2). Following UCL Center for Time Use Research’s recommendations, we excluded participants who did not report the following during the diary day: spending time on sleep/rest/personal care/eating/drinking (*n* = 15); at least one change in location (*n* = 59); and at least one change in whether they spent time in the presence of others (*n* = 13). This left a final analytical sample of 236,270 participants.

The sample was aged 15 and over (mean = 45.14, SD = 18.63), with 52% female, 48% male, 81% identified as White race, 12% Black, 4% Asian, and 2% identified as American Indian, Alaskan Native, Hawaiian/Pacific Islander, or multiple racial groups (Table 1).

Analysis of ATUS data reported in this paper has Institutional Review Board approval from the University of Florida (IRB201901792) and ethical approval from University College London Research Ethics Committee (project 18839/001). All participants in the study gave informed consent.

### Method details

ATUS asked participants to recall their activities over 24 h, beginning at 4a.m. on the day prior to the interview and ending at 4a.m. on the day of the interview. Participants were randomly assigned a day of the week on which to complete the survey, with 10% of the sample allocated to each weekday and 25% to each weekend day. Weights then account for this non-uniform distribution and differing response rates across days of the week, so that measures can be estimated for an average day. Information on secondary activities (activities that are done at the same time as the primary activity) was not collected, except for childcare. Participants reported every activity they took part in during the 24-h period. Activities were coded using a standard lexicon, verified by two coders, and classified within a three-tiered system, from broad to detailed categories including examples.

#### Reading

We focused on two reading outcomes: 1) daily reading for pleasure, classified by ATUS as reading for personal interest (e.g., reading a magazine/book/newspaper, listening to audiobooks, reading on a Kindle or other e-reader; Table S1); and 2) daily reading with children (e.g., reading to or with household or non-household children, listening to child read, helping child read; Table S1). We measured whether participants engaged, and total time (minutes) spent, for each type of reading (reading for personal interest, reading with children; Figure 1). Engagement in each activity was indicated by spending ≥1 min on it during the diary day.

We also measured the social context and location of reading, as ATUS asked participants whom they were with and where they were for every activity. This additional information was used to provide an indication of the types of reading behaviors engaged in. Given the low frequency of reading in the presence of different people, we created a binary indicator of reading alone vs. with others. Similarly, as participants mainly read at home, we measured the proportion of participants who read only in their home/others’ homes vs. outside home.

#### Individual characteristics

Demographic, socioeconomic, and health-related information was collected either during the ATUS interview or from earlier CPS interviews. Measures were based on the data available, with categories determined by ATUS. We selected seven key individual characteristics that have been shown to influence reading.^4^^,^^5^^,^^13^^,^^14^^,^^17^^,^^18^^,^^23^ These were sex (male, female), age group (15–24 years, 25–65 years, 66 years and over), race (White, Black, Asian, Other [including American Indian, Alaskan Native, Hawaiian/Pacific Islander, mixed race]), education (high school or less, college, undergraduate, postgraduate), annual family income (quartiles: less than $30,000, $30,000 - $59,999, $60,000 - $99,999, $150,000 and over), metropolitan area (non-metropolitan area, metropolitan area), and disability status (no disability, any disability that prevents any kind of work). Sex, age, metropolitan area, and disability status were measured in the ATUS interview. Race, education, and income were measured in the CPS interview, 2–5 months before the ATUS interview (mean = 3.04, standard deviation [SD] = 0.57). Further sociodemographic factors were included just in Table 1 to explore the sample characteristics.

### Quantification and statistical analysis

First, we focused on data from 2023, describing current reading practices in the US. We explored the proportion who read (participation rate), average time spent reading overall (total mean), average time spent reading just by participants who read (participation mean),^53^ social context, and activity location for the two types of reading. We describe these measures using percentages for categorical outcomes, and mean and standard deviations for continuous outcomes.

We then aimed to test whether reading behaviors changed from 2003 to 2023. The cross-sectional data collected continuously from January 2003 to December 2023 (excluding 2020) was pooled and survey year used as the time variable. We used a series of regression models to examine time trends. Poisson regression with robust standard errors estimated prevalence ratios, testing whether participation rates changed over time for each activity separately. Robust Poisson regression was chosen over logistic regression because it directly estimates the risk ratio (here referred to as the prevalence ratio), which is more interpretable than the odds ratio when the outcome is common.^54^ Linear regression tested whether the amount of time spent reading (total mean, participation mean) changed over time for each activity. Next, we explored changes in social context and activity location, testing whether the proportion of participants who 1) read alone and 2) read only at home changed over time using Poisson regression with robust standard errors.

Finally, we examined whether reading differed across population groups. Using the data from 2023, we tested whether there were differences in reading for pleasure according to individual characteristics. We tested the association between each individual factor (age, sex, race, education, income, metropolitan area, disability status) and 1) participation rate in separate robust Poisson regression models and 2) participation mean in separate linear regression models. We then pooled data across years and added interactions between individual characteristics and survey year (treated as a linear trend) on these outcomes in separate models, testing whether the disparities between sociodemographic groups changed over the study period. To visualise these interactions, we plotted the association between each characteristic and reading for pleasure stratified by survey year. Given the small group sizes for reading with children (*n* = 200 in 2023), we did not perform analyses limited to 2023, and interactions with time are reported in the supplement (Table S7B).

In all models, time (year) was treated as a linear exposure. We ran two additional sets of models for each outcome to test this assumption: 1) including a quadratic effect of time, and 2) treating time as categorical (the most complex model possible). We compared models using fit statistics (Akaike Information Criterion, Bayesian Information Criterion), Wald tests (quadratic vs. linear model), and likelihood-ratio tests (categorical vs. continuous model). Wald tests indicated that including quadratic terms did not improve model fit (*p* > 0.05), except for reading location. Although likelihood-ratio tests indicated that treating time as categorical did improve model fit over including it as a continuous linear exposure for most outcomes, likelihood-ratio tests are not valid for models with survey weights or clustered data, because the “likelihood” used in this test does not reflect that individual observations are not independent.^55^^,^^56^ Given this, and to avoid overfitting and aid interpretation, time was treated as linear in all models.

ATUS generated weights to account for the two-stage complex sampling – including the sample design and weighting for CPS, oversampling of specific demographic groups from CPS in ATUS (to ensure adequate subgroup sizes), and non-response to ATUS – as well as the non-uniform distribution of the ATUS sample across days of the week, and differing response rates across demographic groups and days of the week. The sum of the weights for weekdays and weekend days is equal to the number of person-days on weekdays and weekend days in the quarter, for selected subpopulations and for the whole population.^25^

We used these weights to generate estimates for an average day representative of the US civilian non-institutionalised population aged 15 and over. Missingness was generally low (<6%; Table S3), although ATUS replaced missing income data using values from previous CPS waves for 9% of participants. To account for data that were still missing, we used multiple imputation by chained equations (MICE).^57^ We generated 20 imputed datasets using ordered logistic and logistic regression according to variable type. The imputation model included all variables used in analyses, sampling weights, and auxiliary variables (e.g., marital status, household size, children in the household, eldercare, employment status, area of residence). Findings from imputed main analyses did not differ to complete case analyses (Tables S8–S11), so imputed results are reported.

In sensitivity analyses, we tested whether outliers in the number of minutes spent reading (participation mean) influenced findings. Top-coding outliers at three standard deviations above the mean or at the 99^th^ percentile did not alter the results, so analyses are reported without outliers removed or recoded. All analyses were performed using Stata MP 18.5.^52^
