# Supplementary material for: The decline in reading for pleasure over 20 years of the American Time Use Survey
Source: iScience. 2025 Aug 20;28(9):113288. doi: 10.1016/j.isci.2025.113288 (PMC12496190; doi:10.1016/j.isci.2025.113288)
Supplement: Document S1. Tables S1–S11 [file mmc1.pdf]

iScience, Volume *28*

Supplemental information

The decline in reading for pleasure over 20 years of the American Time Use Survey

Jessica K. Bone, Feifei Bu, Jill K. Sonke, and Daisy Fancourt

Supplementary Materials

Content	Page
Table S1. ATUS reading activities	2
Table S2. Response rates and analytical sample by year	2
Table S3. Missing data in analytical sample	3
Main analysis results	3
Table S4. Average engagement levels	3
Table S5. Poisson regressions for participation rates	3
Table S6. Linear regressions for time spent on activities	3
Table S7. Interactions with individual characteristics	4
Complete case results	5
Table S8. Sample characteristics	5
Table S9. Average engagement levels	6
Table S10. Poisson regressions for participation rates	6
Table S11. Linear regressions for time spent on activities	6

Table S1. ATUS activity codes for reading activities, with third-tier (detailed) activities shown within the first- and second-tier categories and the examples given to participants for each tier 3 activity as of 2023.

Tier 1	Tier 2	Tier 3	Examples provided (in 2023)
**Reading for pleasure**			
Socialising relaxing and leisure	Relaxing and leisure	Reading for personal interest	reading a magazine/book/newspaper, flipping/leafing through magazine, listening to books on tape/audio books, borrowing books from the library, doing research (personal interest), checking out library books, being read to (personal interest), reading (unspecified), returning library books/browsing at the library, reading a book on a Kindle or other electronic book reader (personal interest)
**Reading to/with children**			
Caring for and helping household members	Caring for and helping household children	Reading to/with household children	reading to or with household child, listening to a household child read, helping household child read
Caring for and helping non-household members	Caring for and helping non-household children	Reading to/with non-household children	reading to or with non-household children, listening to a non-household child read, helping non-household child read

Table S2. Response rates and number of participants in each year for the full analytical sample.

Year	ATUS response rate	Analytical sample size	Proportion of analytical sample
2003	57.8%	20,709	8.8%
2004	57.3%	13,969	5.9%
2005	56.6%	13,036	5.5%
2006	55.1%	12,940	5.5%
2007	52.5%	12,245	5.2%
2008	54.6%	12,719	5.4%
2009	56.6%	13,132	5.6%
2010	56.9%	13,255	5.6%
2011	54.6%	12,476	5.3%
2012	53.2%	12,440	5.3%
2013	49.9%	11,380	4.8%
2014	51.0%	11,586	4.9%
2015	48.5%	10,898	4.6%
2016	46.8%	10,489	4.4%
2017	45.6%	10,217	4.3%
2018	43.0%	9,590	4.1%
2019	42.0%	9,435	4.0%
2021	39.4%	9,081	3.8%
2022	35.8%	8,130	3.4%
2023	36.9%	8,543	3.6%

Table S3. Missing data in the full analytical sample.

Variable	Missing	Proportion
Activity engagement	-	-
Year	-	-
Sex	-	-
Age	-	-
Race	-	-
Education	-	-
Family income	13,693	5.80%
Metropolitan status	1792	0.76%
Disability status	61	0.03%

Note. N=236,270.

Main analysis results

Table S4. Rates of engagement, averaged over time.

Activity	Participation rate	Total mean (SD)	Participation mean (SD)*
Personal interest	21%	18.84 (54.59)	88.46 (88.52)
With children	3%	0.82 (6.18)	30.00 (22.99)

Note. N=236,270. Percentages, means, and standard deviations weighted and based on 20 imputed datasets. *Participation mean calculated only for those who participated in each activity.

Table S5. Poisson regression models testing the associations between survey year and reading.

Outcome	N	Prevalence ratio	95% CI	p value
**Personal interest**				
Participation rate	236,270	0.97	0.97, 0.98	**<0.001**
Reading done with others	56,973	0.99	0.99, 1.00	**<0.001**
Reading done outside home	56,973	0.99	0.98, 0.99	**<0.001**
**With children**				
Participation rate	236,270	1.00	0.99, 1.00	0.211
Reading done with others	8,740*	1.00	1.00, 1.00	0.116
Reading done outside home	8,741	0.98	0.94, 1.01	0.172

Note. Bold text indicates p<0.05. *Social context (with others vs not) of reading with children missing for n=1 and could not be imputed due to convergence issues.

Table S6. Linear regression models testing the associations between survey year and time spent on reading.

Outcome	N	Coefficient	95% CI	P value
**Personal interest**				
Total mean	236,270	-0.36	-0.41, -0.32	**<0.001**
Participation mean	56,973	0.62	0.46, 0.77	**<0.001**
**With children**				
Total mean	236,270	0.00	-0.01, 0.00	0.091
Participation mean	8,741	-0.07	-0.19, 0.05	0.232

Note. Bold text indicates p<0.05.

Table S7a. Tests of whether the associations between individual characteristics and reading for pleasure differed according to survey year (interaction terms from regression models).

	**Participation rate** (n=236,270)			**Participation mean** (n=56,973)		
	Prevalence ratio	95% CI	p value	Coef	95% CI	p value
Sex: Female	1.00	1.00, 1.01	**0.034**	-0.13	-0.45, 0.19	0.431
Age: 25 – 65 years	0.97	0.96, 0.98	**<0.001**	-0.26	-1.05, 0.52	0.508
Age: 66 years and over	0.98	0.97, 0.99	**<0.001**	-0.70	-1.51, 0.11	0.089
Race: Black	0.98	0.98, 0.99	**<0.001**	0.20	-0.40, 0.80	0.511
Race: Asian	1.01	1.00, 1.02	**0.014**	0.39	-0.72, 1.50	0.492
Race: Other	0.99	0.98, 1.01	0.404	0.32	-1.06, 1.71	0.649
Education: College	1.00	1.00, 1.01	0.204	0.22	-0.27, 0.71	0.378
Education: Undergraduate	1.01	1.01, 1.02	**<0.001**	-0.08	-0.52, 0.36	0.724
Education: Postgraduate	1.02	1.01, 1.02	**<0.001**	-0.36	-0.81, 0.10	0.125
Income: $30,000 - $59,999	1.01	1.00, 1.01	**0.015**	-0.04	-0.58, 0.51	0.897
Income: $60,000 - $99,999	1.00	1.00, 1.01	0.340	0.16	-0.35, 0.67	0.540
Income: $100,000 and over	1.01	1.00, 1.01	**0.003**	-0.36	-0.89, 0.17	0.183
Metropolitan status	1.01	1.00, 1.01	**0.007**	-0.65	-1.18, -0.13	**0.015**
Disability status	0.99	0.98, 1.00	0.230	0.62	-1.02, 2.26	0.460

Note. Bold text indicates p<0.05.

Table S7b. Tests of whether the associations between individual characteristics and reading with children differed according to survey year (interaction terms from regression models).

	**Participation rate** (n=236,270)			**Participation mean** (n=8,741)		
	Prevalence ratio	95% CI	p value	Coef	95% CI	p value
Sex: Female	0.98	0.97, 0.99	**0.001**	-0.15	-0.42, 0.11	0.260
Age: 25 – 65 years	1.02	0.99, 1.05	0.229	-0.56	-1.45, 0.33	0.218
Age: 66 years and over	1.06	1.02, 1.11	**0.006**	-0.43	-2.08, 1.22	0.612
Race: Black	1.01	0.99, 1.03	0.417	-0.20	-0.72, 0.32	0.451
Race: Asian	1.01	0.99, 1.02	0.569	-0.44	-0.84, -0.03	**0.035**
Race: Other	1.02	0.98, 1.06	0.427	-1.53	-3.11, 0.05	0.058
Education: College	0.98	0.97, 1.00	**0.036**	-0.16	-0.63, 0.32	0.519
Education: Undergraduate	1.00	0.98, 1.01	0.692	-0.25	-0.69, 0.18	0.257
Education: Postgraduate	1.00	0.98, 1.01	0.946	-0.10	-0.54, 0.34	0.667
Income: $30,000 - $59,999	0.98	0.96, 0.99	**0.012**	-0.03	-0.76, 0.69	0.929
Income: $60,000 - $99,999	0.98	0.97, 1.00	0.072	0.00	-0.69, 0.69	0.998
Income: $100,000 and over	1.00	0.98, 1.01	0.746	0.07	-0.64, 0.76	0.863
Metropolitan status	1.00	0.99, 1.02	0.614	0.08	-0.26,0.41	0.647
Disability status	0.92	0.89, 0.95	**<0.001**	0.10	-4.08, 4.27	0.964

Note. Bold text indicates p<0.05.

Complete case results

Table S8. Characteristics of the complete case sample (weighted).

Characteristic	Proportion
Sex	
Male	48%
Female	52%
Age	
15-24 years	17%
25-65 years	67%
66 years and over	16%
Race	
White	81%
Black	12%
Asian	4%
Other	2%
Marital status	
Married	52%
Widowed/divorced/separated	17%
Never married	31%
Child under 18 in household	40%
Education	
High school or less	45%
College	25%
Undergraduate	19%
Postgraduate	11%
Employment status	
Employed	63%
Unemployed	5%
Not in labour force	17%
Retired	15%
Annual family income	
Less than $30,000	25%
$30,000 - $59,999	28%
$60,000 - $99,999	24%
$100,000 and over	23%
Metropolitan status	
Non-metropolitan area	16%
Metropolitan area	84%
Disability prevents work	4%
	**Mean (SD)**
Household size	2.98 (1.56)
Number of children in household	0.75 (1.13)

Note. N=220,737.

Table S9. Rates of engagement, averaged over time, in complete cases only.

Activity	Participation rate	Total mean (SD)	Participation mean (SD)*
Personal interest	21%	18.59 (54.30)	88.44 (88.63)
With children	3%	0.82 (6.16)	29.85 (22.61)

Note. N=220,737. Percentages, means, and standard deviations weighted and based on 20 imputed datasets. *Participation mean calculated only for those who participated in each activity.

Table S10. Poisson regression models testing the associations between survey year and reading, in complete cases only.

Outcome	N	Prevalence ratio	95% CI	p value
**Personal interest**				
Participation rate	220,737	0.97	0.97, 0.98	**<0.001**
Reading done with others	52,565	0.99	0.99, 1.00	**<0.001**
Reading done outside home	52,565	0.99	0.98, 0.99	**<0.001**
**With children**				
Participation rate	220,737	0.99	0.99, 1.00	**0.025**
Reading done with others	8,314	1.00	1.00, 1.00	0.153
Reading done outside home	8,314	0.98	0.94, 1.02	0.247

Note. Bold text indicates p<0.05.

Table S11. Linear regression models testing the associations between survey year and time spent reading, in complete cases only.

Outcome	N	Coefficient	95% CI	P value
**Personal interest**				
Total mean	220,737	-0.33	-0.38. -0.29	**<0.001**
Participation mean	52,565	0.66	0.49, 0.82	**<0.001**
**With children**				
Total mean	220,737	-0.01	-0.01, 0.00	**0.045**
Participation mean	8,314	-0.03	-0.15, 0.09	0.614

Note. Bold text indicates p<0.05.
